# Development of a school-based programme for mental health promotion and prevention among adolescents in Nepal and South Africa

**DOI:** 10.1016/j.ssmmh.2023.100289

**Published:** 2024-06

**Authors:** Christina A. Laurenzi, Stefani du Toit, Tatenda Mawoyo, Nagendra P. Luitel, Mark J.D. Jordans, Indira Pradhan, Claire van der Westhuizen, G.J. Melendez-Torres, Jemma Hawkins, Graham Moore, Rhiannon Evans, Crick Lund, David A. Ross, Joanna Lai, Chiara Servili, Mark Tomlinson, Sarah Skeen

**Affiliations:** a Institute for Life Course Health Research, Department of Global Health, Faculty of Medicine and Health Sciences, Stellenbosch University, Tygerberg, South Africa; b Research Department, Transcultural Psychosocial Organization Nepal, Baluwatar, Kathmandu, Nepal; c Alan J. Flisher Centre for Public Mental Health, Department of Psychiatry and Mental Health, University of Cape Town, Rondebosch, South Africa; d Center for Global Mental Health, Health Service and Population Research Department, Institute of Psychiatry, Psychology and Neuroscience, King's College London, London, United Kingdom; e University of Exeter, Exeter, United Kingdom; f DECIPHer (Centre for Development, Evaluation, Complexity and Implementation in Public Health Improvement), Cardiff University, Cardiff, Wales, United Kingdom; g Wolfson Centre for Young People's Mental Health, Cardiff University, Cardiff, Wales, United Kingdom; h UNICEF, New York, NY, USA; i Department of Mental Health and Substance Use, World Health Organization, Geneva, Switzerland; j School of Nursing and Midwifery, Queens University, Belfast, Northern Ireland, United Kingdom

**Keywords:** Adolescent mental health, School-based intervention, Psychosocial intervention, Intervention development, Low- and middle-income countries

## Abstract

**Introduction:**

Adolescence is a critical time for mental health promotion and prevention and establishing healthy behaviours. Implementing universal, school-based psychosocial interventions can improve short- and long-term health trajectories for adolescents. While these interventions may offer important opportunities for fostering skills and relationships, few school-based interventions have been developed for and tested in low- and middle-income countries (LMICs) where adolescent mental health needs may be significant and under-served. This manuscript details the development of a multi-component, universal school-based intervention, Health Action in ScHools for a Thriving Adolescent Generation (HASHTAG), for adolescents aged 12–15 years in Nepal and South Africa.

**Methods and results:**

We describe HASHTAG's development over four phases, combining methods and results as each phase was iteratively conducted between 2018 and 2021. Phase 1 included a systematic review and components analysis, building from WHO guidelines for adolescent mental health. Seven components were strongly supported by the evidence: emotional regulation, stress management, mindfulness, problem-solving, interpersonal skills, assertiveness training, and alcohol and drug education. Phase 2 encompassed site selection, theory of change development, and formative research engagements; research teams in each site engaged adolescents and key adult stakeholders to identify priorities for intervention. Stakeholders voiced preferences for external facilitators and key content and delivery for intervention sessions. These findings informed Phase 3, a draft manual of HASHTAG, including a whole-school component, called Thriving Environment in Schools, and a classroom-based, six-session component, Thrive Together. In Phase 4, participants engaged in consultative workshops to review and contextualise content by country, preparing HASHTAG for implementation in a feasibility trial. Minor adaptations were made in Nepal, including using school nurses and adjusting take-home materials; both country's workshops identified practical considerations for implementing activities.

**Conclusions:**

HASHTAG was designed around core evidence-based components to increase translatability across LMICs, while enabling country-specific tailoring to enhance feasibility. Future research will test whether this multi-component, whole-school approach can improve adolescent mental health.

## Highlights

• Seven core components served as a backbone for developing the HASHTAG intervention.
• Multi-stakeholder contributions enhanced the applicability of HASHTAG content.
• HASHTAG was designed to be contextually relevant and adaptable for LMIC settings.

## 1 Introduction

Adolescence is a critical transition period in the life course, accompanied by rapid physical, social, and psychological developmental changes (World Health Organization, 2017). As adolescents enter a period of increased independence and exploration, they are exposed to potential risks to their health and wellbeing that may persist into adulthood (Patton et al., 2016). This is a particularly crucial time for prioritising mental health promotion, given the high burden of disease linked to mental disorders for adolescents and young adults (Mokdad et al., 2016). An estimated 34.6% of all mental health conditions begin before the age of 14 years, and 62.5% by age 25 (Solmi et al., 2022). Furthermore, globally, suicide is the fourth leading cause of death among adolescents and young adults aged 15–29 years (World Health Organization, 2021).

From early adolescence, mental health can profoundly shape individual trajectories. Adolescents managing mental health challenges may have difficulty forming and maintaining interpersonal relationships (Kenny et al., 2013; Li et al., 2020); focusing in school (Cavioni et al., 2021; Juma et al., 2020; Kenny et al., 2013; Schulte-Körne, 2016); and contributing productively to work later in life (Patel et al., 2008). Some adolescents go on to develop chronic mental health conditions or experience more severe episodes of poor mental health (Kessler et al., 2009), and many will remain at risk of developing unhealthy behaviours (such as alcohol, tobacco, and other substance use) and chronic disease into adulthood. Furthermore, despite growing societal awareness and literacy around mental health, stigma linked to poor mental health and lack of access to mental health services persists, compounding the challenges that adolescents and young adults face (Jumbe et al., 2022; Radez et al., 2021; Woodgate et al., 2020).

Engaging adolescents in universal programmes for the promotion of mental health and prevention of mental disorders can promote positive mental health outcomes and prevent or reduce symptoms (World Health Organization, 2020). However, there are significant gaps in the evidence supporting these kinds of interventions. Promotive and preventive interventions have predominantly been implemented and tested in high-income country (HIC) settings (Bonell et al., 2018; Kuyken et al., 2022) despite the fact that approximately 90% of the world's adolescents reside in low- and middle-income countries (LMICs) (Barry et al., 2013; Uppendahl et al., 2020). There has been limited prioritisation of these intervention programmes for adolescent mental health in LMICs (Barbui et al., 2020; Shinde et al., 2018). These programmes also tend to neglect the social and public health context of mental health in the youth population (Laurenzi et al., 2022). Adolescents living in settings with high rates of poverty, conflict, and/or abuse may be more susceptible to poor mental health (Hodgkinson et al., 2017) and may require additional resources, knowledge, and skills to navigate the transition to adulthood.

Across settings, schools are uniquely placed to deliver these kinds of mental health interventions. While a school-based delivery modality is common for universal psychosocial programming in HICs, few studies have evaluated school-based interventions in LMICs, despite their potential for reaching adolescents at scale (Skeen et al., 2019). Beyond providing a convenient, safe, and structured place to engage with the great majority of adolescents, the everyday routines, processes, and practices that adolescents experience at school can support, or conversely, harm, their health and wellbeing. Schools may also be a place where adolescents can apply skills learned to other areas of their lives (King and Fazel, 2019), as well as establish relational skills with peers and teachers (McLaughlin and Clarke, 2010). Supporting mental health in schools may offer additional educational, social, and psychological benefits to adolescents, while also helping to bridge gaps in accessibility (Stephan et al., 2015). Interventions that aim to address individual students’ mental health as well as the broader school climate have also been shown to be valuable (Aldridge and McChesney, 2018). Research suggests that interventions are most effective when they are incorporated into daily practices and the overall culture of schools (Barry et al., 2017; Jones and Bouffard, 2012). This involves engaging all staff members, reinforcing skills beyond just classroom settings, and supporting parental involvement. These findings suggest the potential benefit of incorporating a whole school approach to enhancing the mental health of students (Goldberg et al., 2019). A recent systematic review and meta-analysis on the effectiveness of school-based interventions to improve child and adolescent mental health in LMICs found that interventions delivered in the school environment are effective in improving mental health (Grande et al., 2023). For school staff and policymakers alike in LMIC settings, it may be particularly important to identify intervention approaches that target common causes of multiple behavioural outcomes, in order to increase efficiency of resources.

In response to the need to develop responsive interventions based in school settings in LMICs that target multiple risks to adolescent mental health, we developed a universal, school-based mental health promotion and prevention intervention for adolescents aged 12–15 years. The intervention, named HASHTAG (Health Action in ScHools for a Thriving Adolescent Generation), was based on the input of local advisory boards, teachers and parents, and adolescents themselves, in two diverse contexts—South Africa and Nepal. This paper details the multi-stage development of the HASHTAG intervention.

## 2 Methods and results

### 2.1 Overview of the development of HASHTAG

**Fig. 1:**
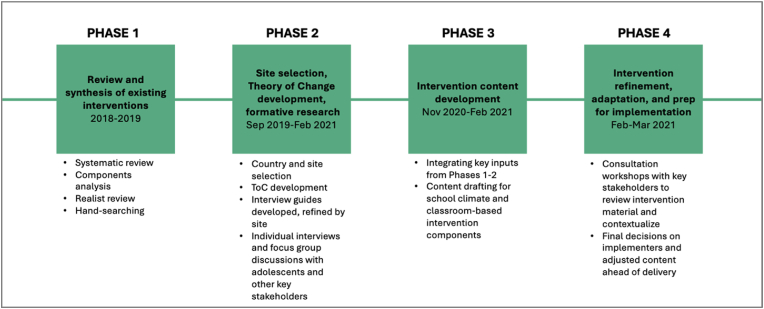
Overview of HASHTAG phases.

HASHTAG was developed iteratively, across two sites in two countries, over a series of four phases, in line with guidance for the development of complex interventions (Skivington et al., 2021). The methods and results are presented in tandem for each of these phases in the sections that follow, to reflect this iterative process. Fig. 1 presents an overview of each phase, with key activities detailed below.

### 2.2 Phase 1: review and synthesis of existing interventions

We began the process of developing the HASHTAG intervention with a review of existing interventions. In 2018–2019, our team conducted a systematic review and meta-analysis of universally delivered psychosocial interventions, including school-based trials, to inform the development of the WHO's Guidelines on Mental Health Promotive and Preventive Interventions for Adolescents, also known as Helping Adolescents Thrive (HAT) (Skeen et al., 2018; World Health Organization, 2020). Psychosocial interventions were defined as interventions that use a psychological, behavioural or social approach, or a combination of these, to improve psychosocial well-being and reduce risk for poor mental health outcomes (England et al., 2015). A related aim was to inform the development of an open-access, universally-delivered intervention to promote adolescent mental health and well-being and to prevent mental health conditions (Skeen et al., 2019).

Of the 158 trials identified in the aforementioned review, n = 111 were delivered in-person in schools; only 10 studies (9.0%) were in LMICs including Brazil, India, Iran, Mexico, South Africa, and Zambia. In addition to conducting a standard meta-analysis to examine the effect of these interventions on specific outcomes, our team also conducted a components analysis. While previous reviews on this topic had typically focused on one outcome only (Calear and Christensen, 2010; Faggiano et al., 2014; Foxcroft and Tsertsvadze, 2011), this components analysis identified intervention content components effective over multiple outcome domains relating to adolescent mental health and risk behaviours. Across outcomes linked to positive mental health, mental health conditions, risk behaviours, and self-harm and suicide, seven components consistently predicted better outcomes when incorporated into psychosocial interventions: assertiveness training; emotional regulation; interpersonal skills; mindfulness; problem-solving skills; stress management; and alcohol and drug education (Skeen et al., 2019).

Following this analysis and in preparation for developing the HASHTAG intervention, we reviewed in depth the interventions that contained one or more of the components identified by the systematic review; we also searched for new studies based in schools in LMIC settings. This revealed one further study with positive outcomes related to mental health in Bihar, India, the SEHER trial (Shinde et al., 2018). Finally, we highlighted one of the effective components— emotional regulation—in a realist review (Skeen et al., 2020). We utilised realist methodologies to examine the contexts and mechanisms through which psychosocial interventions containing emotional regulation strategies operated in practice, and if they worked differently for different groups of adolescents.

Several decisions were made building from the findings of these reviews, the recommendations from the WHO HAT guidelines (World Health Organization, 2020), and the accompanying HAT toolkit (World Health Organization & UNICEF, 2021). We decided to incorporate all seven components in a classroom-based component, alongside school climate considerations from the SEHER trial, into a draft intervention outline. Adolescents between the ages of 12–15 years were selected as the target population, spanning students in Grade 8 in South Africa and Grades 8 and 9 in Nepal. This age range was the most commonly targeted in the existing evidence base (Skeen et al., 2019). Moreover, in both settings, this grade is the first year after transitioning from primary schooling, and is associated with social and educational transitions in each country setting.

### 2.3 Phase 2: site selection, theory of change development, and formative research

In Phase 2, findings from the first phase informed the selection of two diverse low- and/or middle-income countries as the primary sites for developing and implementing HASHTAG. In this section, we detail the study settings and site selection, describe formative research that took place to explore themes of interest for developing the HASHTAG intervention, and outline the development of the intervention's Theory of Change.

#### 2.3.1 Study settings

##### 2.3.1.1 Nepal

Adolescents represent more than one-fifth (22%) of the population of Nepal. They are at high risk of depression and anxiety (Chaulagain et al., 2019). These risks are linked to recent and historical trauma—two major earthquakes in 2015 and a 10-year civil war between 1996 and 2006—against a background of socio-economic deprivation. Nepal is one of the poorest countries in South Asia, ranked 143rd out of the world's 191 countries on the Human Development Index (UNDP, 2022). Just over a quarter of adolescents aged 10–17 years in Nepal are living under multidimensional poverty, as measured across health, education and living standards (National Planning Commission, 2018). A recent nationwide descriptive cross-sectional community-based prevalence study, which included 5,888 adolescent participants, found that the lifetime prevalence of any mental health disorders to be estimated at 5.2% (Dhimal et al., 2021). There is a scarcity of population-wide mental health services for adolescents in Nepal (Luitel et al., 2015), and there is also significant drop-off between primary and secondary education enrolment rates (Ministry of Education Science and Technology, 2020).

We selected the Kanepokhari Rural Municipality in the Morang District in eastern Nepal as the site for HASHTAG. This selection was made because very few school-based mental health interventions had been previously implemented in this area. Morang is the second most populous district in Nepal (population=36,500) (Kanepokhari Rural Municipality, 2019) and is diverse in terms of caste/ethnicity, religion, language and geography.

##### 2.3.1.2 South Africa

Adolescents in South Africa face multiple, overlapping risks that together increase their likelihood of poor mental health. These include high rates of poverty, inequality, unemployment, and substance use; exposure to community and household violence; and a persistent high-prevalence HIV epidemic (Flisher et al., 2012; Lund et al., 2018). Children and adolescents younger than 17 years account for the largest proportion of the poorest population (Statistics South Africa, 2017), and in 2017, 30% of children in South Africa lived in households where no adults were working (Hall, 2019). These circumstances have important implications for adolescents’ ability to navigate the transition to adulthood. While there is limited recent data on the prevalence of mental health disorders in adolescents and young adults in South Africa, data from a large-scale study of 20,855 students in Grade 8–10 in the Western Cape Province found that 14.9% of students were categorised as at high risk of mental health problems based on the mental health subscale of the Problem Oriented Screening Instrument for Teenagers (Plüddemann et al., 2014). Additionally, child and adolescent mental health services across the country are fragmented and extremely limited (Babatunde et al., 2020).

Khayelitsha was the implementation site for the co-development of HASHTAG. Khayelitsha is a large peri-urban area outside of Cape Town, in the Western Cape, and is typical of many of the deeply-entrenched social and economic challenges facing youth in South Africa. Data from South Africa's most recent census indicates that 99.5% of the population of Khayelitsha is Black, with the predominant language being IsiXhosa (Statistics South Africa, 2011). While the official language of education is English, many teachers in settings with other dominant languages utilise a mix of English and the dominant language (a practice sometimes referred to as translanguaging) to ensure student comprehension (Krause and Prinsloo, 2016; Probyn, 2006). Adolescents and young adults aged 15–24 years make up 22% of the population in Khayelitsha (Statistics South Africa, 2011), and there is high enrolment in earlier grades of secondary schools in the area, justifying the selection of this site.

#### 2.3.2 Ethics

Ethical approval for this study was obtained from Stellenbosch University (ref: N19/07/088), University of Cape Town (ref: 265/2020), Queens University Belfast (ref: MTomlinson.SREC_July19_V1), and Nepal Health Research Council (ref: 342/2020P).

Participants either provided written informed consent if aged 18 years or more, or assent if aged less than 18 years. For participants below the age of 18, parents/guardians also provided written consent.

#### 2.3.3 Developing the theory of change

**Fig. 2:**
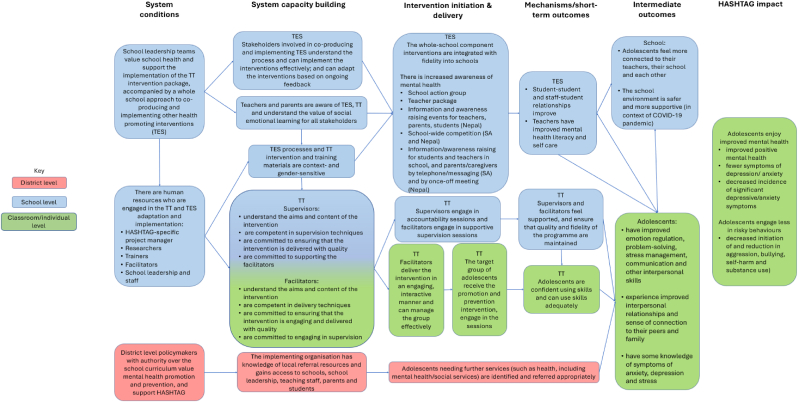
HASHTAG Theory of change.

The HASHTAG Theory of Change (ToC) was developed iteratively. An initial draft was based on the Helping Adolescents Thrive (HAT) ToC, which was intended to inform the implementation, monitoring and evaluation of HAT programming, and which was developed in consultation with the South African team from the University of Cape Town and Stellenbosch University, in collaboration with WHO and UNICEF colleagues and international stakeholders during a series of face-to-face workshops and online meetings in 2019. Prior to fieldwork activities, the HASHTAG team contextualised the HAT ToC for South Africa and Nepal. The HASHTAG ToC map (Fig. 2) hypothesised causal pathways, including outcomes necessary for implementation, actions to achieve these outcomes, and indicators to measure whether the outcomes were achieved. Later iterations incorporated findings from the formative qualitative work, described below, in each country. The ToC was revised as necessary throughout the phases of the study as lessons were learned from formative research engagements in both countries.

#### 2.3.4 Formative research: methods

Following site selection, formative research activities were conducted. Each country team held focus group discussions (FGDs) with adolescents, and conducted key informant interviews with caregivers/parents, teachers, and key community stakeholders. Because this phase was conducted during the COVID-19 pandemic, research teams in each country had to adjust research methods to adhere to COVID-19 protocols—including shifting from more collaborative approaches to largely individual approaches that protected participants’ safety. Further, due to the ongoing pandemic and timing of engagements, government representatives were interviewed in Nepal only.

Draft interview guides were circulated within the study team and refined; questions included challenges experienced by adolescents at school, the impact of school-related problems on adolescents, and questions about acceptable group intervention characteristics (for example, ideal venue, time of intervention, and facilitators). Participants for the key informant interviews and FGDs were recruited through existing community networks, which included established adolescent advisory boards, community organisations working with children, youth, and families, and schools within the selected sites. All participants across both countries provided written consent to participate and be recorded, and adolescent participants were asked to provide written assent as well as have their parent or legal guardian consent.

Formative research engagements included semi-structured individual interviews and FGDs. These engagements were conducted across both countries with school-going adolescents, teachers, parents/caregivers of school-going adolescents, and other community-based and governmental representatives.

Table 1 shows which stakeholders were included across both countries, and details which data collection method was used for each group.

**Table 1:** Participants in formative research engagements.

	Nepal	South Africa
Adolescents (ages 12–15)	Not applicable	n = 12, across 3 focus groups
Adolescents (ages 12–18)	n = 47, across 5 focus groups	Not applicable
Parents/caregivers of school-going adolescents	n = 25 (n = 19 across 2 focus groups, n = 6 individual interviews)	n = 12 individual interviews
Teachers	n = 8 individual interviews	n = 8 individual interviews
NGO representatives	n = 3 individual interviews	n = 4 individual interviews
School management representatives	n = 4 individual interviews	n = 2 individual interviews
Community leaders, members of youth clubs	n = 5 individual interviews	Not applicable
Government representatives	n = 7 individual interviews	Not applicable

In each country, a small team of research assistants with expertise in qualitative research and working with adults and adolescents conducted the research engagements. All participants consented to be audio-recorded. In Nepal, research engagements were conducted in Nepali, between December 2020–January 2021. All interviews and FGDs were conducted in person, in private rooms within selected school areas. On average, the interviews took approximately 60 min, while FGDs lasted for approximately 90 min. In South Africa, research engagements were conducted in IsiXhosa, between November 2020–February 2021. Some participants were interviewed remotely; however, all FGDs took place in-person.

Audio recordings from research engagements in both countries were transcribed and translated, enabling thematic analysis of transcripts. In each country, two researchers familiarised themselves with the data, developed a coding frame, and applied codes to transcripts using both traditional and rapid analysis techniques to populate key themes and emerging insights, organised by participant type (Suchman et al., 2023).

#### 2.3.5 Formative research: findings

In Nepal, most stakeholders supported the idea of a school-based mental health intervention. Teachers and students suggested that gender-mixed groups would be better, while parents felt boys and girls should be in separate groups, reasoning that girls could feel hesitant to open up in a mixed-gender group. A number of participants suggested that parents also should be targeted by the intervention because parents have important roles in improving the psychosocial wellbeing of their children. Participants suggested that an external facilitator, preferably a mental health expert, conduct sessions.

In South Africa, stakeholders explained a range of challenges experienced by adolescents; emerging themes included risky alcohol and drug use, bullying, and a strained relationship with teachers. In addition, participants also suggested key characteristics of the facilitators that would deliver the adolescent group intervention. The most common characteristics included being bilingual (IsiXhosa and English), friendly, energetic, patient and mature. Regarding the skills to be taught during the intervention, participants cited assertiveness, empathy, and expression of feelings as essential skills.

### 2.4 Phase 3: intervention content development

Building from Phases 1 and 2, the structure and content for the HASHTAG intervention was developed. It consisted of two intervention components: 1) a school climate component, Thriving Environment in School (TES), and 2) a classroom-based group intervention, Thrive Together (TT). All intervention content was designed to be delivered in resource-limited contexts by trained individuals without specialist mental health training, which could include teachers, school nurses, or lay facilitators.

#### 2.4.1 Thriving Environment in Schools

The first part, *Thriving Environment in Schools (TES)*, was conceptualised as a whole-school approach to promoting mental health in schools. It was designed as a set of activities to improve adolescents' social and emotional environment to create a school culture of connectedness. Each of these provisions, as articulated in the ToC, were designed to improve student-student and staff-student relationships, and improve teachers’ mental health literacy and self-care capabilities. TES consisted of three core activities (see Table 2).

**Table 2:** Overview of the generic HASHTAG intervention package (Thriving Environment in Schools component).

Dimension	Key components	Goal
Mental health awareness	• A school-wide awareness campaign, designed to include: • school-wide launch of HASHTAG; • a school-wide competition for students to enter in teams, to promote mental health	• To raise awareness among all students in the intervention school and provide opportunities for reinforcing key messages.
School Action Groups	• Group that meets regularly and includes school staff (teachers and/or school officials) and student representatives (from Grades 8–12)	• To implement whole-school activities, especially around mental health awareness, and to establish sustainable in-school infrastructure that could support broader school climate improvements.
Teacher wellbeing modules	Module 1: Getting to know one another	• To build rapport between facilitators and participants; to introduce HASHTAG; to introduce self-care
	Module 2: Understanding stress and self-care	• To increase teachers' stress management skills
	Module 3: Tips and tools for teachers of adolescents	• To improve teachers' connection and reflective engagement with adolescents
	Follow-up messages disseminated after modules	• To reinforce key messages from modules. An example includes: • *Caring for others comes naturally in most of us, however it is always important to take care of yourself first, so you have enough to share with others.*

##### 2.4.1.1 Mental health awareness

Conducting mental health awareness for all school stakeholders was seen as crucial for the successful implementation of TES. The first activity involved a school-wide launch, which would include informing students, teachers, and other school personnel about HASHTAG. At this launch, a school-wide competition was announced; this competition was to be designed with student leadership (see more information regarding School Action Groups below) and was integrated into the launch programme to build excitement. The competition was formulated to enable students to creatively express their views on what mental health means to them and how to improve mental health.

##### 2.4.1.2 School Action Groups

It was seen as important for schools to have mechanisms prior to project inception to organise HASHTAG activities and offer guidance on how to adapt them to the specific workings of each school. School Action Groups for each school were part of the TES portfolio, with representatives from both teachers and students. Launch events were identified as a place for School Action Groups to be introduced, with representatives later chosen from each school grade to meet on a weekly basis.

##### 2.4.1.3 Teacher wellbeing modules

The objectives and content of the teacher wellbeing modules were based on the needs of teaching staff identified during the formative process (see details above). Some of these needs included enhancing the relationship between teachers and adolescents, identified as a key theme during the formative stage. Once the core needs were established, the intervention development team searched for existing teacher self-care modules suitable for low-resource settings that did not require a high level of expertise and resources to deliver. Two existing programmes were identified that matched these criteria, both unpublished: (1) CORE for teachers, developed by War Child (intervention designed to support teachers in war-affected areas); and (2) the Zihoye curriculum (self-care intervention designed for frontline workers in the South African context). The final teacher wellbeing modules consisted of three modules that cover key concepts of self-care, emotional wellbeing, interpersonal skills, and classroom management (see Table 2). Follow-up messages were devised to disseminate to teachers following these modules.

#### 2.4.2 Thrive Together

The second part of the intervention, *Thrive Together* (TT), comprised a group-based intervention programme for adolescents. The structure and content for TT was based on findings from the systematic review and meta-analysis conducted in Phase 1, which found seven effective programme components, as well as the emerging findings from the formative research conducted during Phase 2. In line with the ToC, it was hypothesised that adolescents would experience increased confidence in using skills taught in these sessions—leading to improved intermediate outcomes linked to core component-based skills, interpersonal relationships, connection to peers, and mental health knowledge.

##### 2.4.2.1 Programme structure

The programme consisted of six sessions, to be delivered on a weekly basis. Each session was planned to last between 60 and 90 min. The duration of the programme and length of the sessions enabled the programme to be delivered during the school day with minimal disruptions to the school curriculum. All sessions followed a similar structure, starting with an “emotional check-in,” a recap of the previous session; additionally, all sessions included an affirmation activity. The main content of the specific session followed and each session ended with a mindfulness activity. The intervention utilised a combination of delivery components, including group work, role-playing, group discussions, and individual activities.

##### 2.4.2.2 Session content

Certain sessions centred around core components: emotional regulation, problem-solving, stress management, interpersonal skills, and assertiveness training. Two other components, mindfulness and alcohol and drug use, were integrated as cross-cutting features, woven into multiple sessions. The order of the sessions was determined in a way that enabled key themes to build on one another over time (see Table 3), and take-home comics were developed to accompany each session and be shared post-session with students. Comics were based on findings from the review and formative phases, and were developed by members of the HASHTAG research team (including JL) with partners at UNICEF. Additionally, reinforcing critical messages for intervention session participants were proposed, so that specific messages echoing themes and takeaways from each session could be distributed to students and caregivers via SMS (South Africa) or once-off school meeting (Nepal) following the sessions to which they were tied. All content, including case studies, was designed to be culturally and gender sensitive and appropriate, and was framed by the findings of the focus groups.

**Table 3:** Overview of the manualised HASHTAG intervention package (Thrive Together component).

Session	Title	Goal	Content Components	Delivery component	Activities included	Sample key messages disseminated to students/parents after sessions
Session 1	Introduction to Thrive Together	To introduce students to “Thriving Together”, establish an open supportive environment, and discuss introductory concepts	Emotional regulation, mindfulness	Group discussions; psychoeducation; visuals	• Feeling thermometer • My feelings (pleasant and unpleasant feelings) • Mindfulness	• *As much as we would like to always experience positive/pleasant feelings, it is also fine to experience unpleasant feelings.* • *A deep breathing exercise can help you identify your current feelings. Practice it so you can understand your body.*
Session 2	Taking Control of my Feelings	To learn skills to take care of mental and physical health	Stress management; emotional regulation; mindfulness	Group discussions; vignette; visuals; psychoeducation	• Feeling thermometer • How my feelings affect my body • Affirmation • Mindfulness	• *Practicing mindfulness meditation, thinking about your favourite place, can help you improve your feelings.* • *Some feelings are too much to take, sometimes you need to share with a trusted adult.*
Session 3	Coping with my Stress	To improve students' understanding of stress and its impact on their minds and bodies. To develop students' helpful coping strategies to deal with stress.	Stress management; drug and alcohol knowledge; Mindfulness	Group discussions; psychoeducation; visuals	• Feeling thermometer • My body and stress • Helpful and unhelpful coping strategies • Affirmation • Mindfulness exercise	• *A lot of things can cause us stress: some of them are within our control, some are not.* • *Remember we cannot control all our stressors, but we can learn how to manage them better so that they do not overwhelm us.*
Session 4	Taking Charge	To introduce strategies for solving problems and for students to identify which strategies work for them.	Problem solving; mindfulness	Group discussions; psychoeducation; visuals; group work	• Feeling thermometer • 5 Step Problem Solving • Affirmation • Mindfulness exercise	• *When we feel overwhelmed and hopeless, we won't always attempt to solve problems, but when we have a clear formula of solving a problem, we will feel confident to try.*
Session 5	Relationships	To improve students' understanding of the importance of relationships as a way to promote wellbeing, and to practice skills that improve the quality of interpersonal relationships.	Interpersonal skills; mindfulness	Group discussions; psychoeducation; role play; group work; visuals	• Feeling thermometer • Interpersonal skills • Practice active listening • Affirmation • Mindfulness exercise	• *Remember, having good relationships in our lives, such as family and friends, can enrich our lives.* • *Skills that help you communicate and interact well with others are extremely important for good relationships.*
Session 6	Difficult Conversations	To learn and improve assertiveness skills to use in difficult situations	Assertiveness training; drug and alcohol knowledge	Group discussions; psychoeducation; role play; visuals	• Feeling thermometer • Introduction to assertiveness • Standing up to peer pressure • Affirmation • Mindfulness exercise	• *Being true to yourself and those around you at all times is important* • *Peer pressure can be avoided by being assertive*

##### 2.4.2.3 Manualisation and material development planning

As the intervention content was mapped and session-based content developed, an intervention manual was drafted to standardise each session across sites, led by the South Africa research team. The study team also developed a student workbook, to guide each participant through sessions while also providing a private space for them to reflect. These workbooks included a calendar and an “about me” section, and participants could add photos and write about themselves and their hobbies.

### 2.5 Phase 4: intervention refinement, adaptation, and preparation for implementation

Phase 4 was the final stage in developing HASHTAG, and enabled teams in each site to review and contextualise the intervention package (TES and TT) emerging from Phase 3. Table 4 details emerging findings from this process across both countries, with additional details by country presented below.

**Table 4:** Consultation sessions structure and findings.

Site	HASHTAG aspect under review	Which groups reviewed this aspect?	Themes of questions asked during consultation	Key findings
Nepal	School Action Groups	• Teachers and stu\dents	• Thoughts about the concept of groups • Composition of the group • Ideas around recruitment • Frequency of meetings	• All grades should be represented in the School Action Groups • Composition to include school management committee members and the school principal, and include more students than adults • SAG meetings should be conducted on Fridays as school dismisses early • Meetings on a fortnightly or monthly basis
	School-wide activities	Students	• Activities to promote school-wide mental health awareness	• Including debate competition, theatre, dancing competition, and singing competition among students
	Teacher wellbeing modules	Students	• Activities to include in teacher training and structure of training	• Provide training to all teachers across multiple grade levels • Include a topic on ‘treating all students equally’
	TT sessions	• Counsellors • Psychological experts	• Adjustments to activities to make them more culturally appropriate • Adjustments to improve clarity	• Shift in terminology from “emotional regulation” to “emotional management” • Facilitators to demonstrate activities where possible (e.g. deep breathing activity) • Change terminology from “feelings thermometer” to “wheel of emotion” to better describe emotions, with definition of mental health and mental illness • Play slow music during meditation • Affirmation activities should integrate culturally appropriate framing
South Africa	School Action Groups	• Adolescent advisory board • Grade 9 representatives • Teachers and school management representatives	• Thoughts about the idea • Composition of the group • Ideas around recruitment • Potential barriers of effectively engaging with the group • Frequency of meetings	• All grades should be represented in the School Action Groups • Facilitators should present the School Action Group idea in a fun and creative way • School Action Group meetings should be conducted on Fridays as school dismisses early
	School-wide competition	• Adolescent advisory board • Grade 9 representatives • Teachers and school management representatives	• Thoughts about the idea • Incentives for the competition • Best ways to market the competition • Support needed from school staff members to make the competition a success	• Use of posters to market the competition • Support for the idea of having incentives to encourage participation • Creative expression to be used for instance, drawing and poetry
	Teacher wellbeing modules	• Adolescent advisory board • Grade 9 representatives	• Thoughts about the idea • What makes it difficult to relate to teachers from a student perspective • Skills needed by teachers to connect with adolescents • Way to engage teachers to make teacher modules successful • Sharing of characteristics of teachers that have had a positive impact on the adolescents	• Importance of teachers being approachable • Importance of teachers building rapport with students and keeping sensitive information confidential • Avoidance of favouritism
		• Teachers and school management representatives	• Thoughts about the idea • Ideal venue for sessions • Stressors in the role due to COVID-19	• Appreciation for willingness to consider teacher well-being • Offsite venue preferred for teacher wellbeing modules • Additional responsibilities accumulated due to COVID-19

#### 2.5.1 Consultation workshops and adaptation: Nepal

The generic HASHTAG intervention package was adapted for the Nepali context using multiple methods, including a thorough review of the intervention manual by psychologists and psychosocial counsellors and a consultative workshop with mental health experts (n = 11 participants). Four additional workshops were conducted among teachers (n = 19) and students (n = 17) in Kanepokhari Rural Municipality. In each workshop, an overview of the HASHTAG intervention was presented, and teachers' and students’ perceptions and feedback were collected.

Additional instructions for facilitators were added to help with preparation and logistics. Terminology and cultural specificity was discussed in more detail, with several changes suggested in terms of how activities were framed (Table 4). These consultations also resulted in the study team deciding to remove the take-home comics, because the images and stories included in the comics were not viewed as relevant for the Nepali context, and cultural adaptation of the images and content was not possible in the short time period ahead of planned implementation. As additional referral resources, and in light of limited professional child mental health services, contact numbers were added to intervention materials for nearby health facilities and helpline phone numbers (Child Helpline 1098, Kanti Hospital's toll-free number, the Transcultural Psychosocial Organization's Helpline, and Suicide Prevention helpline).

#### 2.5.2 Consultation workshops and adaptation: South Africa

A series of three once-off workshops, with diverse stakeholders, took place in South Africa after the formative interviews and FGDs (between February–March 2021) and following selection of study schools. Three groups were established to explore and contextualise ideas regarding HASHTAG's whole-school TES component, as well as to discuss the relevance and acceptability of specific activities.

The three groups included: 1) Adolescent Advisory Board; 2) Grade 9 representatives; 3) teachers and representatives of school management. The Adolescent Advisory Board workshop group was comprised of 5 members who are routinely engaged for adolescent-related matters. The Grade 9 representatives' group (n = 8) comprised 2 students per school who had completed Grade 8 at that same school; each school was represented by one male and one female participant. Teachers (n = 7) and school management representatives (n = 2) comprised a mix of representatives from the four selected study schools in Khayelitsha (same schools as the Grade 9 representatives).

In each workshop, general ideas were presented and facilitators asked for participants’ thoughts about specific aspects. For instance, all three groups were asked about the School Action Groups: specifically, facilitators probed for general thoughts about the concept of these groups, reflections on their ideal composition, and when and where meetings should ideally take place. The same was done for teacher wellbeing modules, and the school-wide competition. While most of the consultations were discussion-based, material for one of the self-care exercises was piloted with a group of teachers and representatives from school management. The information obtained during the consultations provided guidance for the development and adaptation of the final content (see Table 4).

#### 2.5.3 From development to implementation

From these workshops, minor adjustments enabled appropriate tailoring by country. Workshop findings encouraged the study team in each country to make final-stage adjustments to the manuals and materials to be used in implementation, including decisions on framing and key lessons to emphasise. They also dictated some important decisions about how the resulting intervention would be delivered. Most significantly, it was determined that school nurses would deliver the TT intervention in Nepal, while external lay facilitators would do so in South Africa. Final optimisation decisions—for instance, around how to integrate HASHTAG into school infrastructure and ensure alignment with school policies—were made in the early stage of implementation and will be detailed in a forthcoming feasibility paper. The results of a pilot feasibility trial testing HASHTAG, conducted in both countries following Phase 4, will be detailed in this same forthcoming publication.

## 3 Discussion

In response to a gap in available psychosocial prevention and promotion interventions tailored for adolescents from LMICs, we developed HASHTAG to be delivered to young adolescents in schools to promote positive mental health. Prioritising feasibility in the intervention's ultimate delivery, especially in resource-limited contexts, was a primary consideration during the development process.

### 3.1 Strengths

The resulting intervention, HASHTAG, has several strengths for delivery in a resource-limited context. The intervention was designed around core components with an aim to increase its translatability across settings, and especially for LMIC settings. Based on a rigorous review of the literature, and a principal components analysis, all components integrated into the TT sessions were independently shown to be effective, and recommended by WHO and UNICEF through the HAT toolkit (World Health Organization & UNICEF, 2021). The intervention was then carefully contextualised through formative research and adaptation workshops in Nepal and South Africa, which largely validated the proposed content and design. To this end, the involvement of a wide range of stakeholders, from diverse settings in Nepal and South Africa, were crucial to the development and implementation processes. The process of developing the intervention with ongoing, structured input from community stakeholders ensured that the intervention was able to speak to the current, specific needs of adolescents and teachers; that its components were relevant to the educational context; and that it was culturally sensitive. Additionally, the aim of HASHTAG was to meaningfully include both teachers and students to improve school climate, and allow students to engage throughout the intervention implementation and governance as part of the School Action Group leadership—something uncommon among similar interventions. While this multi-stage, multi-actor engagement may have presented opportunities to reinforce hierarchies between students and teachers in their school setting, we found that being aware of diverse perspectives enabled us to build common understandings, through formative stages as well as when School Action Groups took shape.

Furthermore, while the team initially planned to develop country-specific theories of change to complement the generic HASHTAG ToC, major adaptations due to country differences were not required. Two notable differences in programmatic structure are worth discussing. Different cadres of stakeholders were recruited for the formative work, due to variations in the administration processes of local government and services. Additionally, stakeholders in Nepal recommended school nurses as intervention facilitators, while South African stakeholders preferred external lay facilitators. Although the subsequent adaptation processes in Nepal and South Africa resulted in diverse applications of key content, this contextualisation enabled teachers and students in each setting to have more ownership of and connection to the intervention.

Secondly, decisions about HASHTAG's delivery were made with an aim to increase feasibility. Its individual psychosocial intervention component, TT, uses a group-based approach, delivering content to classroom units. This approach is less labour intensive than individually-delivered interventions, and can be less expensive to deliver (Lopez Garcia et al., 2021). Group formats further provide adolescents with opportunities to practice behavioural modelling, role-playing, group problem-solving, and reinforcing behaviours with peers to enhance social competence (Martel, 2018), meaning there are both universal and context-specific aspects to their implementation. Additionally, the use of non-specialist implementers, for TT in particular but also for school-wide activities, can increase an intervention's applicability and appeal for resource-limited settings. Because there is a significant shortage of trained mental health professionals in LMIC settings, task-shifting to non-specialists is a practical and effective strategy (Barbui et al., 2020; Malik et al., 2021; Oyedeji et al., 2022). For instance, the cluster randomised trial conducted in India referenced above, SEHER, that examined the efficacy of a multi-component school climate intervention, found that the intervention was more successful when provided by lay counsellors (young women with a Master's degree in psychology or a related subject), in comparison to conventional life skills delivered by teachers; these effects were measured by increases in school climate evaluations and moderate to significant decreases in bullying and depression (Shinde et al., 2018). While deploying teachers to deliver HASHTAG's TT sessions may be able to enhance its scalability and sustainability in resource-limited settings, considerations around the pedagogical culture of the school, teacher burdens, and school climate more broadly, are critical to ensure fidelity and quality. It is likely more plausible that integrating teachers into school-climate components—such as extending the roles that they can play within TES activities—may be a more suitable way to both enhance sustainability of these interventions while also not creating additional burdens on teachers operating in resource-limited settings. Because funding is particularly important in LMIC settings when considering school-based interventions, low-cost approaches (group settings, non-specialist implementers) can be attractive to policymakers.

Finally, this group-based intervention was also designed for delivery against the backdrop of a school climate component. Research suggests that a positive school climate is crucial for healthy social, mental, emotional, and behavioural outcomes (Lester and Cross, 2015). Improvements in school climate have been found to improve adolescents’ mental health as well as their academic performance, school satisfaction, sense of belonging, behaviour, and overall positive health and wellbeing (Bond et al., 2004; Lester and Cross, 2015; Wang et al., 2010; Zullig et al., 2010). In addition, positive school climate has also been found to serve as a protective factor against teacher burnout (Cohen et al., 2009; Grayson and Alvarez, 2008).

### 3.2 Limitations

While the two-country development and adaptation processes were a strength of HASHTAG, these two processes and resulting interventions also played out somewhat distinctly as the intervention package took shape. Additionally, because this study coincided with the first year of the COVID-19 pandemic, and because of differing peaks in COVID-19 infections and lockdowns across Nepal and South Africa, the development and contextualisation of HASHTAG happened on slightly different timelines. Context-specific adaptations, while key for supporting uptake in each site, can pose challenges for comparability in evaluation. Even when interventions are kept more standardised, however, they may differ in the implementation phase, whether or not these differences are well-documented. An upcoming publication of the evaluation of HASHTAG in both sites will examine the question of potential differences more closely.

While we engaged several diverse stakeholders, including adolescents themselves, across both sites, our development processes did not reflect as many provisions of true co-development as would have been ideal. Again as a result of the COVID-19 pandemic, changes in face-to-face research practices, to prioritise participant and staff safety, prevented us from engaging stakeholders as robustly as we had originally envisioned, such as through workshops or more power-equitable approaches. Engaging with policymakers more substantively during the early and later stages of HASHTAG development may have also established foundations for later sustainability, something to consider in the next stages of implementation.

A final limitation involves the inclusion of adolescents’ networks—specifically their parents and caregivers. While more comprehensive parent engagement may have strengthened lessons from HASHTAG in the home environment, the current project resources, and the complex contextual considerations linked to the COVID-19 pandemic, shaped the ultimate decision to focus on school-based delivery only.

### 3.3 Implications and future direction

In Nepal, the National Mental Health Strategy and Action Plan of the Government of Nepal targets include mental health promotion and prevention activities in the school curriculum in coordination with the Ministry of Education, as well as the provision of training on child and adolescent mental health to teachers and school health workers (MoHP, 2020). The HASHTAG intervention could be instrumental in supporting the government strategy to prevent mental health problems among school-going adolescents in Nepal. Second, in the formative research, both teachers and students recommended an external delivery agent (not teachers) to implement the HASHTAG intervention. Recently, the government of Nepal has started the “One School, One Nurse” programme in selected districts. Training school nurses to deliver school-based mental health promotion programmes could be a viable and sustainable strategy to scale up school-based mental health activities throughout Nepal. In the forthcoming publication describing the pilot trial of HASHTAG, we will share findings on feasibility and acceptability of using trained nurses to implement HASHTAG's TT intervention sessions.

In South Africa, there is a minimum package of services that all secondary schools should be implementing to support students’ mental health—including promoting a safe school environment and protecting against discrimination and bullying, identifying and supporting children who may be experiencing abuse, and disaster risk reduction (Skeen et al., 2022). Schools are mandated to establish school-based support teams, which may employ Learner Support Agents who are tasked with supporting learners experiencing barriers to learning; alongside other school-based and community partners. For many students, however, these policies and provisions may not be sufficient to safeguard their mental health. Poor implementation of policies is coupled with limited resource availability and multiple, overlapping adversities facing students—including substance use, adolescent pregnancy, and violence at home. For students in these situations, who are facing several chronic stressors in the context of developmental transitions through adolescence and school, supportive programming such as HASHTAG may be critical to enhancing this school-based support and potentially providing a means to referral to specialist care where needed. Greater awareness of adolescent mental health needs by teachers, school personnel, and caregivers—as well as adolescents themselves—also has the potential to expand avenues for advocacy and accountability.

The newly developed HASHTAG intervention aims to address the scarcity of research-grounded universal mental health prevention programmes in LMICs. Especially in contexts such as the COVID-19 pandemic, humanitarian crises and other adversities, mental health prevention programmes can help buffer the impact of disrupted schooling, social isolation, and caregiver loss. Future evaluation research will test whether this multi-component, whole-school approach, is effective in achieving this aim.

## CRediT authorship contribution statement

**Christina A. Laurenzi:** Conceptualization, Data curation, Formal analysis, Funding acquisition, Investigation, Methodology, Project administration, Supervision, Writing – original draft, Writing – review & editing. **Stefani du Toit:** Investigation, Methodology, Project administration, Supervision, Writing – original draft, Writing – review & editing. **Tatenda Mawoyo:** Formal analysis, Investigation, Methodology, Project administration, Supervision, Writing – original draft, Writing – review & editing. **Nagendra P. Luitel:** Conceptualization, Data curation, Funding acquisition, Investigation, Project administration, Supervision, Writing – original draft. **Mark J.D. Jordans:** Conceptualization, Funding acquisition, Investigation, Methodology, Project administration, Supervision, Writing – original draft. **Indira Pradhan:** Investigation, Methodology, Project administration, Writing – original draft. **Claire van der Westhuizen:** Conceptualization, Funding acquisition, Investigation, Validation, Visualization, Writing – original draft. **G.J. Melendez-Torres:** Conceptualization, Data curation, Formal analysis, Funding acquisition, Methodology, Writing – original draft. **Jemma Hawkins:** Funding acquisition, Investigation, Methodology, Writing – original draft, Writing – review & editing. **Graham Moore:** Conceptualization, Formal analysis, Funding acquisition, Investigation, Methodology, Writing – original draft, Writing – review & editing. **Rhiannon Evans:** Conceptualization, Formal analysis, Funding acquisition, Investigation, Methodology, Writing – original draft, Writing – review & editing. **Crick Lund:** Conceptualization, Funding acquisition, Writing – original draft. **David A. Ross:** Conceptualization, Data curation, Funding acquisition, Investigation, Methodology, Writing – original draft, Writing – review & editing. **Joanna Lai:** Conceptualization, Investigation, Methodology, Project administration, Writing – original draft. **Chiara Servili:** Conceptualization, Funding acquisition, Investigation, Writing – original draft. **Mark Tomlinson:** Conceptualization, Funding acquisition, Investigation, Resources, Writing – original draft. **Sarah Skeen:** Conceptualization, Formal analysis, Funding acquisition, Investigation, Methodology, Project administration, Supervision, Writing – original draft, Writing – review & editing.

## Declaration of competing interest

The authors declare that they have no known competing financial interests or personal relationships that could have appeared to influence the work reported in this paper.
